# (11a*S*)-1,5,11,11a-Tetra­hydro-1-benzo­thieno[3,2-*f*]indolizin-3(2*H*)-one

**DOI:** 10.1107/S1600536813031693

**Published:** 2013-11-23

**Authors:** Viktor Vrábel, Július Sivý, Peter Šafář, Jozef Kožíšek

**Affiliations:** a Institute of Analytical Chemistry, Faculty of Chemical and Food Technology, Slovak Technical University, Radlinského 9, SK-812 37 Bratislava, Slovak Republic; b Institute of Mathematics and Physics, Faculty of Mechanical Engineering, Slovak University of Technologyy, Námestie slobody 17, SK-812 31 Bratislava, Slovak Republic; c Institute of Organic Chemistry, Catalysis and Petrochemistry, Faculty of Chemical and Food Technology, Slovak Technical University, Radlinského 9, SK-812 37 Bratislava, Slovak Republic; d Institute of Physical Chemistry and Chemical Physics, Slovak University of Technology, Radlinského 9, SK-812 37 Bratislava, Slovak Republic

## Abstract

The absolute configuration of the title compound, C_14_H_13_NOS, was assigned from the synthesis and confirmed by the structure determination. There are two independent mol­ecules in the asymmetric unit. The central six-membered ring of the indolizine moiety adopts an envelope conformation, with the greatest deviations from the mean planes being 0.569 (3) and 0.561 (3) Å for the indolizine bridgehead C atoms of the two mol­ecules. The benzothieno ring attached to the indolizine ring system is planar to within 0.015 (3) Å in both mol­ecules. In the crystal, weak C—H⋯O and C—H⋯π inter­actions lead to the formation of a three-dimensional framework structure.

## Related literature

For background to indolizine derivatives, see: Gubin *et al.* (1992 ▶); Gupta *et al.* (2003 ▶); Liu *et al.* (2007 ▶); Medda *et al.* (2003 ▶); Molyneux & James (1982 ▶); Nash *et al.* (1988 ▶); Pearson & Guo (2001 ▶); Ruprecht *et al.* (1989 ▶); Smith *et al.* (2007 ▶); Teklu *et al.* (2005 ▶). For ring conformations, see: Cremer & Pople (1975 ▶). For the synthesis, see: Šafář *et al.* (2009 ▶). For a related structure, see: Vrábel *et al.* (2012 ▶).
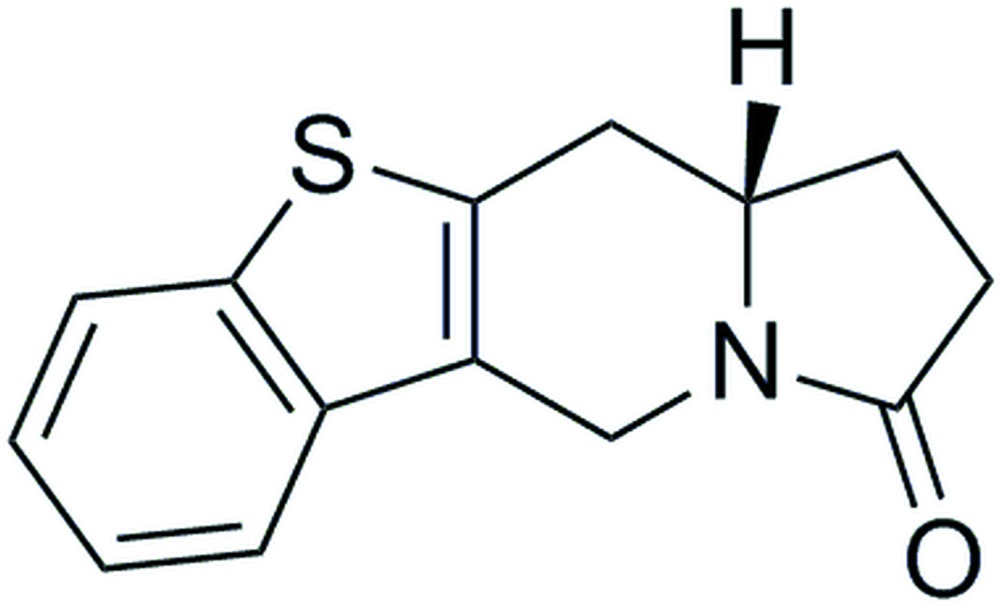


## Experimental

### 

#### Crystal data

C_14_H_13_NOS
*M* *_r_* = 243.31
Monoclinic, 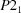

*a* = 9.3327 (8) Å
*b* = 12.4575 (7) Å
*c* = 10.3103 (7) Å
β = 105.469 (8)°
*V* = 1155.27 (14) Å^3^
*Z* = 4
Mo *K*α radiation
μ = 0.26 mm^−1^
*T* = 295 K
0.30 × 0.20 × 0.15 mm

#### Data collection

Oxford Diffraction Xcalibur (Ruby, Gemini) diffractometer
Absorption correction: analytical (*CrysAlis RED*; Oxford Diffraction, 2009 ▶) *T*_min_ = 0.942, *T*_max_ = 0.969
17520 measured reflections
4061 independent reflections
2918 reflections with *I* > 2σ(*I*)
*R*_int_ = 0.093

#### Refinement

*R*[*F* ^2^ > 2σ(*F* ^2^)] = 0.040
*wR*(*F* ^2^) = 0.065
*S* = 0.94
4061 reflections
307 parameters
1 restraint
H-atom parameters constrained
Δρ_max_ = 0.19 e Å^−3^
Δρ_min_ = −0.16 e Å^−3^
Absolute structure: Flack (1983 ▶), 1923 Friedel pairs
Absolute structure parameter: −0.07 (6)

### 

Data collection: *CrysAlis CCD* (Oxford Diffraction, 2009 ▶); cell refinement: *CrysAlis CCD*; data reduction: *CrysAlis RED* (Oxford Diffraction, 2009 ▶); program(s) used to solve structure: *SHELXS97* (Sheldrick, 2008 ▶); program(s) used to refine structure: *SHELXL97* (Sheldrick, 2008 ▶); molecular graphics: *PLATON* (Spek, 2009 ▶), *WinGX* (Farrugia, 2012 ▶) and *DIAMOND* (Brandenburg, 2001 ▶); software used to prepare material for publication: *SHELXL97*.

## Supplementary Material

Crystal structure: contains datablock(s) I, global. DOI: 10.1107/S1600536813031693/bq2390sup1.cif

Structure factors: contains datablock(s) I. DOI: 10.1107/S1600536813031693/bq2390Isup2.hkl

Supplementary material file. DOI: 10.1107/S1600536813031693/bq2390Isup3.cml

Additional supplementary materials:  crystallographic information; 3D view; checkCIF report

## Figures and Tables

**Table 1:** Hydrogen-bond geometry (Å, °) *Cg*4 and *Cg*14 are the centroids of the C8–C13 and C22–C27 rings, respectively.

*D*—H⋯*A*	*D*—H	H⋯*A*	*D*⋯*A*	*D*—H⋯*A*
C20—H20*B*⋯O2^i^	0.97	2.48	3.307 (4)	144
C3—H3*B*⋯*Cg*14	0.97	2.59	3.502 (3)	157
C17—H17*A*⋯*Cg*4	0.97	2.92	3.800 (4)	151
C29—H29*B*⋯*Cg*4^ii^	0.97	2.90	3.706 (3)	142

Symmetry codes: (i) 
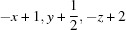
; (ii) 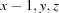.

**Fig. 1.**
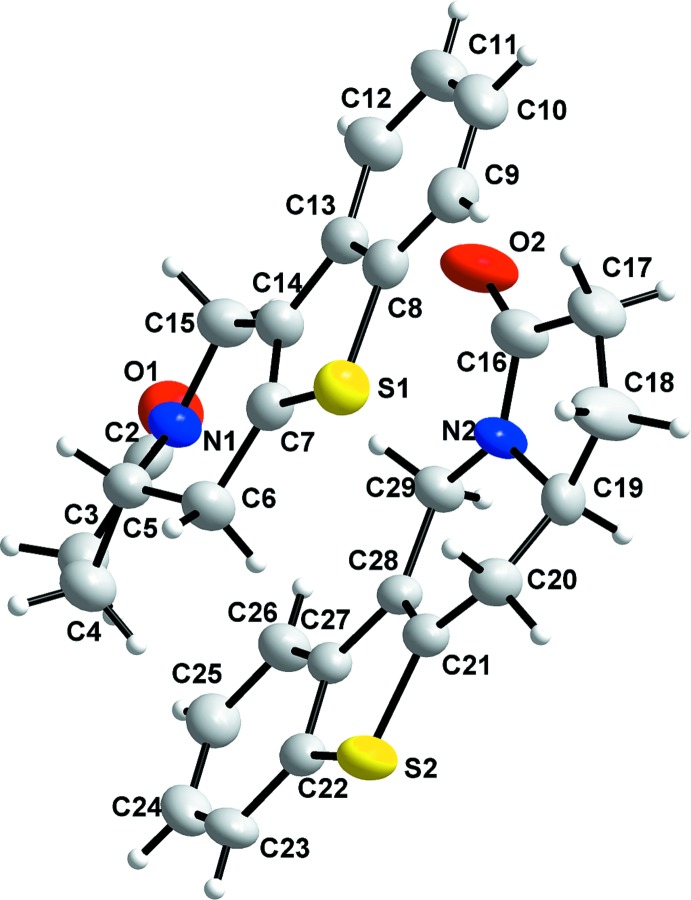
Molecular structure of the title compound showing the atom labeling scheme of the two independent molecules. Displacement ellipsoids are drawn at the 50% probability level (Brandenburg, 2001).

**Fig. 2.**
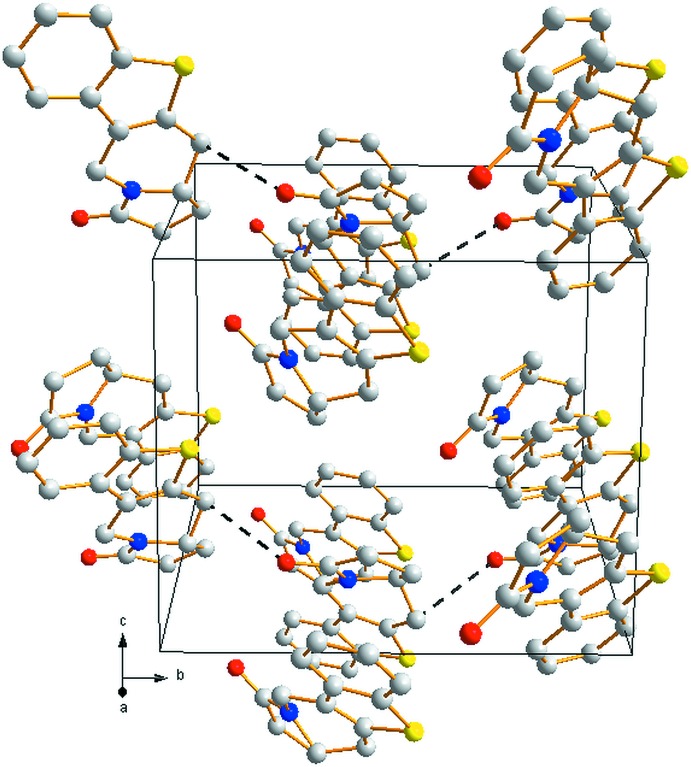
Molecular packing view of the title compound (I) in the crystal structure. Hydrogen bonds were shown as dashed lines. H atoms have been omitted for clarity.

## supplementary crystallographic information

### 1. Comment

Heterocycles are involved in a wide range of biologically important chemical reactions in living organisms, and therefore they form one of the most important and well investigated classes of organic compounds. One group of heterocycles, indolizines, has received much scientific attention during the recent years. Indolizine derivatives have been found to possess a variety of biological activities such as antibacterial, anti-inflammatory, antiviral, (Nash *et al.*, 1988; Molyneux & James, 1982; Medda *et al.*, 2003), anti-HIV (Ruprecht *et al.*, 1989), anti-cancer (Liu *et al.*, 2007; Smith *et al.*, 2007), and antitumor (Pearson & Guo, 2001). They have also shown to be calcium entry blockers (Gupta *et al.*, 2003) and potent antioxidants inhibiting lipid peroxidation in vitro (Teklu *et al.*, 2005). As such, indolizines are important synthetic targets in view of developing new pharmaceuticals for the treatment of cardiovascular diseases (Gubin *et al.*, 1992). Based on these facts and in continuation of our interest in developing simple and efficient route for the synthesis of novel indolizine derivatives, we report here the synthesis, molecular and crystal structure of the title compound (I), which crystallizes in the monoclinic space group P21 with two crystallographic independent molecules in asymmetric unit. The absolute configuration has been established without ambiguity from the anomalous dispersion of the S atom (Flack, 1983) and assigned consistent with the starting material. The expected stereochemistry of both atoms C5 and C19 was confirmed as S, see Fig. 1. Molecular packing view of the title compound (I) in the crystal structure is shown in Fig. 2. The central six-membered N-heterocyclic ring is not planar and assumes a chair conformation, with total puckering amplytude QT of 0.406 (3) Å and orientation angles theta and phi of 0.129,5 (5)° and 169 (5)° (QT of 0.400 (3) Å, theta and phi of 173,3 (5)° and 128,8 (4)°, respectively, for second molecule) (Cremer & Pople, 1975). Atoms C5 and C19 are displaced of 0.569 (3) Å and -0.561 (3) Å, respectively, from the C9/C10/C12/C13 and C20/C21/C28/C29/N2 mean planes. The dihedral angles between the planes of the central N-heterocyclic ring and the plane of the pyrrolidine ring are 24.7 (1)° and 24.1 (1)°, respectively, for second molecule. Atoms N1 and N2 are *sp*^2^-hybridized, as evidenced by the sum of the valence angles around them (358.54° and 358.99°, respectively, for second molecule). These data are consistent with conjugation of the lone-pair electrons on nitrogen atom with the adjacent carbonyl, similar to what is observed for amides. Bond lengths and angles in the indolizine ring system are in good agreement with values from the literature (Vrábel *et al.*, 2012). The molecular structure is stabilized by weak intramolecular C–H···O interactions (Fig.2). The molecular packing is further stabilized by C–H···Pi interactions [C3–H3B···*Cg*14i: *Cg*14 are the centroid of the rings defined by the atoms C22—C27; C17–H17A···*Cg*4i and C29–H29B···*Cg*4ii: *Cg*4 are the centroid of the rings defined by the atoms C8—C13; symmetry operator for generating equivalent atoms: (i) *x*, *y*, *z*; (ii) -1 + *x*, *y*, *z*].

### 2. Experimental

The title compound was prepared according to a standard protocol described in literature (Šafář *et al.*, 2009).

### 3. Refinement

All H atoms were positioned with idealized geometry using a riding model with C—H distances are in the range 0.93 - 0.98 Å. The *U*_iso_(H) values were set at 1.2 *U*_eq_(C-aromatic). An absolute structure was established using anomalous dispersion effects; 1923 Friedel pairs were not merged.

### Crystal data

**Table:**

C_14_H_13_NOS	*F*(000) = 512
*M**_r_* = 243.31	*D*_x_ = 1.399 Mg m^−^^3^
Monoclinic, *P*2_1_	Mo *K*α radiation, λ = 0.71073 Å
Hall symbol: P 2yb	Cell parameters from 3929 reflections
*a* = 9.3327 (8) Å	θ = 3.9–24.6°
*b* = 12.4575 (7) Å	µ = 0.26 mm^−^^1^
*c* = 10.3103 (7) Å	*T* = 295 K
β = 105.469 (8)°	Block, colourless
*V* = 1155.27 (14) Å^3^	0.30 × 0.20 × 0.15 mm
*Z* = 4	

### Data collection

**Table:**

Oxford Diffraction Xcalibur (Ruby, Gemini) diffractometer	4061 independent reflections
Radiation source: fine-focus sealed tube	2918 reflections with *I* > 2σ(*I*)
Graphite monochromator	*R*_int_ = 0.093
Detector resolution: 10.4340 pixels mm^-1^	θ_max_ = 25.0°, θ_min_ = 3.8°
ω scans	*h* = −11→11
Absorption correction: analytical (*CrysAlis RED*; Oxford Diffraction, 2009)	*k* = −14→14
*T*_min_ = 0.942, *T*_max_ = 0.969	*l* = −12→12
17520 measured reflections	

### Refinement

**Table:**

Refinement on *F*^2^	Secondary atom site location: difference Fourier map
Least-squares matrix: full	Hydrogen site location: inferred from neighbouring sites
*R*[*F*^2^ > 2σ(*F*^2^)] = 0.040	H-atom parameters constrained
*wR*(*F*^2^) = 0.065	*w* = 1/[σ^2^(*F*_o_^2^) + (0.0158*P*)^2^] where *P* = (*F*_o_^2^ + 2*F*_c_^2^)/3
*S* = 0.94	(Δ/σ)_max_ < 0.001
4061 reflections	Δρ_max_ = 0.19 e Å^−^^3^
307 parameters	Δρ_min_ = −0.16 e Å^−^^3^
1 restraint	Absolute structure: Flack (1983), 1923 Friedel pairs
Primary atom site location: structure-invariant direct methods	Absolute structure parameter: −0.07 (6)

### Special details

**Table:**

Geometry. All e.s.d.'s (except the e.s.d. in the dihedral angle between two l.s. planes) are estimated using the full covariance matrix. The cell e.s.d.'s are taken into account individually in the estimation of e.s.d.'s in distances, angles and torsion angles; correlations between e.s.d.'s in cell parameters are only used when they are defined by crystal symmetry. An approximate (isotropic) treatment of cell e.s.d.'s is used for estimating e.s.d.'s involving l.s. planes.
Refinement. Refinement of *F*^2^ against ALL reflections. The weighted *R*-factor *wR* and goodness of fit *S* are based on *F*^2^, conventional *R*-factors *R* are based on *F*, with *F* set to zero for negative *F*^2^. The threshold expression of *F*^2^ > σ(*F*^2^) is used only for calculating *R*-factors(gt) *etc*. and is not relevant to the choice of reflections for refinement. *R*-factors based on *F*^2^ are statistically about twice as large as those based on *F*, and *R*- factors based on ALL data will be even larger.

### Fractional atomic coordinates and isotropic or equivalent isotropic displacement parameters (Å^2^)

**Table:**

	*x*	*y*	*z*	*U*_iso_*/*U*_eq_	
C9	1.1545 (4)	0.5063 (2)	1.0070 (3)	0.0510 (9)	
H9	1.1930	0.5751	1.0058	0.061*	
C10	1.2105 (4)	0.4396 (3)	1.1129 (3)	0.0575 (9)	
H10	1.2865	0.4632	1.1853	0.069*	
C11	1.1545 (4)	0.3363 (3)	1.1131 (3)	0.0549 (9)	
H11	1.1951	0.2907	1.1850	0.066*	
C12	1.0399 (3)	0.3006 (2)	1.0084 (3)	0.0510 (8)	
H12	1.0030	0.2314	1.0105	0.061*	
C15	0.7720 (3)	0.2472 (2)	0.7527 (3)	0.0493 (8)	
H15B	0.8362	0.1895	0.7391	0.059*	
H15A	0.7339	0.2284	0.8286	0.059*	
C2	0.5277 (4)	0.1948 (2)	0.6049 (3)	0.0460 (8)	
C3	0.4370 (3)	0.2215 (2)	0.4647 (3)	0.0505 (8)	
H3B	0.3350	0.2374	0.4640	0.061*	
H3A	0.4372	0.1619	0.4042	0.061*	
C4	0.5101 (4)	0.3191 (2)	0.4224 (3)	0.0560 (9)	
H4B	0.4530	0.3834	0.4271	0.067*	
H4A	0.5183	0.3109	0.3311	0.067*	
C6	0.7111 (3)	0.4385 (2)	0.5692 (3)	0.0483 (8)	
H6B	0.7476	0.4757	0.5018	0.058*	
H6A	0.6266	0.4781	0.5826	0.058*	
C8	1.0392 (3)	0.4704 (2)	0.9009 (3)	0.0402 (7)	
C13	0.9791 (3)	0.36689 (19)	0.8999 (3)	0.0386 (7)	
C7	0.8309 (3)	0.43324 (19)	0.6986 (3)	0.0410 (7)	
C14	0.8593 (3)	0.3489 (2)	0.7827 (3)	0.0392 (7)	
C5	0.6634 (3)	0.3257 (2)	0.5214 (3)	0.0443 (8)	
H5	0.7373	0.2940	0.4808	0.053*	
C23	0.0755 (3)	0.3963 (2)	0.4371 (3)	0.0459 (8)	
H23	0.0459	0.4457	0.3675	0.055*	
C24	0.0128 (3)	0.2967 (2)	0.4263 (3)	0.0490 (8)	
H24	−0.0590	0.2776	0.3483	0.059*	
C25	0.0553 (3)	0.2236 (2)	0.5311 (3)	0.0489 (8)	
H25	0.0109	0.1562	0.5228	0.059*	
C26	0.1622 (3)	0.2495 (2)	0.6473 (3)	0.0440 (8)	
H26	0.1891	0.2003	0.7174	0.053*	
C29	0.4260 (3)	0.3311 (2)	0.8896 (3)	0.0419 (7)	
H29B	0.3558	0.3157	0.9416	0.050*	
H29A	0.4627	0.2634	0.8649	0.050*	
C16	0.6609 (4)	0.3507 (2)	1.0638 (3)	0.0497 (8)	
C17	0.7553 (4)	0.4401 (2)	1.1378 (3)	0.0576 (9)	
H17B	0.7465	0.4451	1.2292	0.069*	
H17A	0.8589	0.4287	1.1406	0.069*	
C18	0.6977 (4)	0.5393 (3)	1.0606 (3)	0.0731 (10)	
H18B	0.7623	0.5611	1.0057	0.088*	
H18A	0.6915	0.5976	1.1213	0.088*	
C20	0.5094 (3)	0.5562 (2)	0.8317 (3)	0.0439 (7)	
H20B	0.4790	0.6306	0.8323	0.053*	
H20A	0.5974	0.5533	0.7987	0.053*	
C22	0.1835 (3)	0.42259 (19)	0.5527 (3)	0.0365 (7)	
C27	0.2297 (3)	0.3498 (2)	0.6591 (2)	0.0338 (7)	
C21	0.3872 (3)	0.4920 (2)	0.7414 (3)	0.0375 (7)	
C28	0.3499 (3)	0.3920 (2)	0.7654 (2)	0.0338 (6)	
C19	0.5440 (4)	0.51079 (19)	0.9726 (3)	0.0466 (8)	
H19	0.4683	0.5343	1.0165	0.056*	
N1	0.6489 (3)	0.25973 (16)	0.6329 (2)	0.0427 (6)	
N2	0.5487 (3)	0.39360 (16)	0.9700 (2)	0.0430 (6)	
O1	0.5026 (3)	0.12637 (15)	0.6797 (2)	0.0613 (6)	
O2	0.6828 (3)	0.25454 (16)	1.0830 (2)	0.0706 (7)	
S1	0.94828 (9)	0.54148 (6)	0.75781 (7)	0.0501 (2)	
S2	0.28209 (8)	0.54190 (6)	0.58804 (7)	0.0476 (2)	

### Atomic displacement parameters (Å^2^)

**Table:**

	*U*^11^	*U*^22^	*U*^33^	*U*^12^	*U*^13^	*U*^23^
C9	0.044 (2)	0.0506 (19)	0.055 (2)	−0.0032 (15)	0.0087 (18)	−0.0123 (14)
C10	0.043 (2)	0.074 (2)	0.049 (2)	−0.0014 (19)	0.0014 (16)	−0.0114 (17)
C11	0.048 (2)	0.071 (2)	0.042 (2)	0.0015 (18)	0.0059 (17)	0.0063 (15)
C12	0.049 (2)	0.0541 (18)	0.0468 (19)	−0.0069 (16)	0.0081 (18)	0.0042 (14)
C15	0.048 (2)	0.0450 (17)	0.049 (2)	0.0015 (15)	0.0036 (17)	0.0027 (13)
C2	0.036 (2)	0.0430 (18)	0.057 (2)	0.0069 (15)	0.0098 (17)	−0.0096 (16)
C3	0.039 (2)	0.0594 (19)	0.050 (2)	0.0049 (16)	0.0052 (16)	−0.0099 (15)
C4	0.052 (2)	0.061 (2)	0.047 (2)	0.0021 (17)	0.0016 (17)	−0.0016 (15)
C6	0.055 (2)	0.0417 (16)	0.0427 (18)	0.0017 (15)	0.0038 (16)	0.0023 (13)
C8	0.040 (2)	0.0402 (17)	0.0414 (18)	0.0004 (14)	0.0134 (16)	−0.0031 (12)
C13	0.0358 (19)	0.0418 (17)	0.0387 (17)	0.0004 (13)	0.0112 (15)	−0.0003 (13)
C7	0.043 (2)	0.0371 (16)	0.0429 (18)	0.0020 (13)	0.0110 (15)	−0.0021 (13)
C14	0.043 (2)	0.0348 (15)	0.0384 (17)	−0.0012 (13)	0.0079 (15)	0.0008 (12)
C5	0.044 (2)	0.0455 (17)	0.0397 (18)	0.0064 (14)	0.0040 (15)	−0.0017 (13)
C23	0.035 (2)	0.0551 (19)	0.0393 (18)	0.0062 (15)	−0.0044 (15)	0.0019 (14)
C24	0.0348 (19)	0.064 (2)	0.0426 (19)	−0.0037 (16)	−0.0001 (15)	−0.0093 (15)
C25	0.039 (2)	0.0517 (18)	0.053 (2)	−0.0080 (15)	0.0067 (17)	−0.0070 (15)
C26	0.044 (2)	0.0436 (17)	0.0400 (18)	−0.0026 (14)	0.0039 (15)	0.0021 (13)
C29	0.041 (2)	0.0412 (16)	0.0375 (17)	−0.0023 (14)	0.0008 (15)	0.0007 (13)
C16	0.047 (2)	0.054 (2)	0.043 (2)	−0.0011 (16)	0.0025 (17)	0.0058 (15)
C17	0.049 (2)	0.065 (2)	0.048 (2)	−0.0036 (18)	−0.0049 (16)	−0.0054 (16)
C18	0.064 (2)	0.0528 (18)	0.076 (2)	−0.009 (2)	−0.0280 (19)	−0.011 (2)
C20	0.0431 (19)	0.0358 (15)	0.0482 (17)	−0.0014 (14)	0.0043 (15)	−0.0004 (14)
C22	0.0300 (18)	0.0410 (16)	0.0378 (17)	0.0039 (13)	0.0077 (14)	−0.0015 (13)
C27	0.0271 (17)	0.0387 (15)	0.0341 (16)	0.0020 (12)	0.0056 (13)	−0.0019 (12)
C21	0.0327 (18)	0.0419 (15)	0.0363 (17)	0.0032 (13)	0.0064 (15)	−0.0005 (12)
C28	0.0286 (17)	0.0362 (15)	0.0347 (16)	0.0042 (12)	0.0048 (14)	0.0002 (12)
C19	0.048 (2)	0.046 (2)	0.0414 (18)	0.0017 (14)	0.0059 (16)	−0.0128 (12)
N1	0.0402 (16)	0.0426 (13)	0.0399 (15)	−0.0010 (12)	0.0013 (13)	0.0031 (10)
N2	0.0426 (17)	0.0377 (13)	0.0371 (14)	−0.0006 (11)	−0.0098 (13)	0.0006 (11)
O1	0.0606 (16)	0.0558 (13)	0.0660 (16)	−0.0128 (12)	0.0143 (12)	0.0043 (11)
O2	0.0664 (18)	0.0542 (14)	0.0728 (16)	−0.0012 (12)	−0.0136 (13)	0.0196 (11)
S1	0.0572 (6)	0.0374 (4)	0.0534 (5)	−0.0046 (4)	0.0106 (4)	0.0005 (4)
S2	0.0444 (5)	0.0419 (4)	0.0493 (4)	0.0005 (4)	0.0001 (4)	0.0096 (4)

### Geometric parameters (Å, º)

**Table:**

C9—C10	1.361 (4)	C23—C24	1.364 (4)
C9—C8	1.389 (4)	C23—C22	1.380 (3)
C9—H9	0.9300	C23—H23	0.9300
C10—C11	1.389 (4)	C24—C25	1.387 (4)
C10—H10	0.9300	C24—H24	0.9300
C11—C12	1.375 (4)	C25—C26	1.378 (4)
C11—H11	0.9300	C25—H25	0.9300
C12—C13	1.385 (4)	C26—C27	1.389 (3)
C12—H12	0.9300	C26—H26	0.9300
C15—N1	1.454 (3)	C29—N2	1.449 (3)
C15—C14	1.493 (4)	C29—C28	1.495 (3)
C15—H15B	0.9700	C29—H29B	0.9700
C15—H15A	0.9700	C29—H29A	0.9700
C2—O1	1.214 (3)	C16—O2	1.222 (3)
C2—N1	1.358 (4)	C16—N2	1.333 (3)
C2—C3	1.505 (4)	C16—C17	1.497 (4)
C3—C4	1.514 (4)	C17—C18	1.490 (4)
C3—H3B	0.9700	C17—H17B	0.9700
C3—H3A	0.9700	C17—H17A	0.9700
C4—C5	1.522 (4)	C18—C19	1.520 (4)
C4—H4B	0.9700	C18—H18B	0.9700
C4—H4A	0.9700	C18—H18A	0.9700
C6—C7	1.496 (4)	C20—C21	1.497 (4)
C6—C5	1.516 (3)	C20—C19	1.512 (3)
C6—H6B	0.9700	C20—H20B	0.9700
C6—H6A	0.9700	C20—H20A	0.9700
C8—C13	1.405 (3)	C22—C27	1.399 (3)
C8—S1	1.736 (3)	C22—S2	1.735 (3)
C13—C14	1.428 (4)	C27—C28	1.443 (4)
C7—C14	1.343 (3)	C21—C28	1.333 (3)
C7—S1	1.742 (3)	C21—S2	1.739 (3)
C5—N1	1.449 (3)	C19—N2	1.461 (3)
C5—H5	0.9800	C19—H19	0.9800
			
C10—C9—C8	119.1 (3)	C23—C24—H24	119.8
C10—C9—H9	120.5	C25—C24—H24	119.8
C8—C9—H9	120.5	C26—C25—C24	120.9 (3)
C9—C10—C11	120.3 (3)	C26—C25—H25	119.5
C9—C10—H10	119.9	C24—C25—H25	119.5
C11—C10—H10	119.9	C25—C26—C27	119.5 (3)
C12—C11—C10	120.8 (3)	C25—C26—H26	120.2
C12—C11—H11	119.6	C27—C26—H26	120.2
C10—C11—H11	119.6	N2—C29—C28	109.9 (2)
C11—C12—C13	120.5 (3)	N2—C29—H29B	109.7
C11—C12—H12	119.8	C28—C29—H29B	109.7
C13—C12—H12	119.8	N2—C29—H29A	109.7
N1—C15—C14	110.4 (2)	C28—C29—H29A	109.7
N1—C15—H15B	109.6	H29B—C29—H29A	108.2
C14—C15—H15B	109.6	O2—C16—N2	125.2 (3)
N1—C15—H15A	109.6	O2—C16—C17	126.6 (3)
C14—C15—H15A	109.6	N2—C16—C17	108.2 (2)
H15B—C15—H15A	108.1	C18—C17—C16	105.4 (2)
O1—C2—N1	125.1 (3)	C18—C17—H17B	110.7
O1—C2—C3	127.7 (3)	C16—C17—H17B	110.7
N1—C2—C3	107.2 (3)	C18—C17—H17A	110.7
C2—C3—C4	105.8 (3)	C16—C17—H17A	110.7
C2—C3—H3B	110.6	H17B—C17—H17A	108.8
C4—C3—H3B	110.6	C17—C18—C19	105.8 (3)
C2—C3—H3A	110.6	C17—C18—H18B	110.6
C4—C3—H3A	110.6	C19—C18—H18B	110.6
H3B—C3—H3A	108.7	C17—C18—H18A	110.6
C3—C4—C5	105.4 (2)	C19—C18—H18A	110.6
C3—C4—H4B	110.7	H18B—C18—H18A	108.7
C5—C4—H4B	110.7	C21—C20—C19	109.3 (2)
C3—C4—H4A	110.7	C21—C20—H20B	109.8
C5—C4—H4A	110.7	C19—C20—H20B	109.8
H4B—C4—H4A	108.8	C21—C20—H20A	109.8
C7—C6—C5	109.5 (2)	C19—C20—H20A	109.8
C7—C6—H6B	109.8	H20B—C20—H20A	108.3
C5—C6—H6B	109.8	C23—C22—C27	121.6 (2)
C7—C6—H6A	109.8	C23—C22—S2	127.6 (2)
C5—C6—H6A	109.8	C27—C22—S2	110.82 (19)
H6B—C6—H6A	108.2	C26—C27—C22	118.5 (2)
C9—C8—C13	121.7 (3)	C26—C27—C28	129.5 (2)
C9—C8—S1	127.3 (2)	C22—C27—C28	111.9 (2)
C13—C8—S1	111.0 (2)	C28—C21—C20	125.3 (3)
C12—C13—C8	117.7 (2)	C28—C21—S2	113.0 (2)
C12—C13—C14	130.5 (2)	C20—C21—S2	121.7 (2)
C8—C13—C14	111.8 (2)	C21—C28—C27	112.9 (2)
C14—C7—C6	125.6 (2)	C21—C28—C29	123.0 (3)
C14—C7—S1	112.4 (2)	C27—C28—C29	124.1 (2)
C6—C7—S1	121.96 (19)	N2—C19—C20	110.9 (2)
C7—C14—C13	113.5 (2)	N2—C19—C18	102.5 (3)
C7—C14—C15	121.8 (2)	C20—C19—C18	114.4 (3)
C13—C14—C15	124.7 (2)	N2—C19—H19	109.6
N1—C5—C6	110.4 (2)	C20—C19—H19	109.6
N1—C5—C4	103.4 (2)	C18—C19—H19	109.6
C6—C5—C4	114.2 (2)	C2—N1—C5	114.7 (2)
N1—C5—H5	109.5	C2—N1—C15	122.8 (2)
C6—C5—H5	109.5	C5—N1—C15	121.0 (2)
C4—C5—H5	109.5	C16—N2—C29	123.2 (2)
C24—C23—C22	119.0 (3)	C16—N2—C19	114.1 (2)
C24—C23—H23	120.5	C29—N2—C19	121.6 (2)
C22—C23—H23	120.5	C8—S1—C7	91.27 (13)
C23—C24—C25	120.4 (3)	C22—S2—C21	91.34 (13)
			
C8—C9—C10—C11	1.2 (5)	C19—C20—C21—C28	−21.4 (4)
C9—C10—C11—C12	−1.4 (5)	C19—C20—C21—S2	160.9 (2)
C10—C11—C12—C13	0.6 (5)	C20—C21—C28—C27	−178.7 (3)
O1—C2—C3—C4	175.0 (3)	S2—C21—C28—C27	−0.9 (3)
N1—C2—C3—C4	−7.1 (3)	C20—C21—C28—C29	1.1 (4)
C2—C3—C4—C5	15.9 (3)	S2—C21—C28—C29	179.0 (2)
C10—C9—C8—C13	−0.1 (5)	C26—C27—C28—C21	179.2 (3)
C10—C9—C8—S1	178.7 (2)	C22—C27—C28—C21	2.2 (3)
C11—C12—C13—C8	0.4 (4)	C26—C27—C28—C29	−0.7 (5)
C11—C12—C13—C14	−178.5 (3)	C22—C27—C28—C29	−177.7 (2)
C9—C8—C13—C12	−0.7 (4)	N2—C29—C28—C21	−4.3 (4)
S1—C8—C13—C12	−179.7 (2)	N2—C29—C28—C27	175.6 (3)
C9—C8—C13—C14	178.4 (3)	C21—C20—C19—N2	43.4 (3)
S1—C8—C13—C14	−0.6 (3)	C21—C20—C19—C18	158.6 (3)
C5—C6—C7—C14	−20.3 (4)	C17—C18—C19—N2	−19.3 (3)
C5—C6—C7—S1	161.2 (2)	C17—C18—C19—C20	−139.3 (3)
C6—C7—C14—C13	−179.1 (3)	O1—C2—N1—C5	172.6 (3)
S1—C7—C14—C13	−0.5 (3)	C3—C2—N1—C5	−5.3 (3)
C6—C7—C14—C15	1.1 (5)	O1—C2—N1—C15	6.3 (5)
S1—C7—C14—C15	179.8 (2)	C3—C2—N1—C15	−171.7 (2)
C12—C13—C14—C7	179.6 (3)	C6—C5—N1—C2	137.8 (3)
C8—C13—C14—C7	0.7 (4)	C4—C5—N1—C2	15.3 (3)
C12—C13—C14—C15	−0.7 (5)	C6—C5—N1—C15	−55.6 (3)
C8—C13—C14—C15	−179.6 (3)	C4—C5—N1—C15	−178.1 (2)
N1—C15—C14—C7	−6.2 (4)	C14—C15—N1—C2	−159.5 (3)
N1—C15—C14—C13	174.1 (3)	C14—C15—N1—C5	35.0 (4)
C7—C6—C5—N1	43.3 (3)	O2—C16—N2—C29	8.5 (5)
C7—C6—C5—C4	159.2 (3)	C17—C16—N2—C29	−172.7 (3)
C3—C4—C5—N1	−18.4 (3)	O2—C16—N2—C19	177.2 (3)
C3—C4—C5—C6	−138.4 (2)	C17—C16—N2—C19	−4.1 (4)
C22—C23—C24—C25	1.1 (4)	C28—C29—N2—C16	−160.2 (3)
C23—C24—C25—C26	−0.6 (5)	C28—C29—N2—C19	31.9 (3)
C24—C25—C26—C27	−0.7 (5)	C20—C19—N2—C16	137.3 (3)
O2—C16—C17—C18	169.8 (3)	C18—C19—N2—C16	14.9 (3)
N2—C16—C17—C18	−8.9 (4)	C20—C19—N2—C29	−53.8 (3)
C16—C17—C18—C19	17.6 (4)	C18—C19—N2—C29	−176.3 (3)
C24—C23—C22—C27	−0.2 (4)	C9—C8—S1—C7	−178.7 (3)
C24—C23—C22—S2	178.3 (2)	C13—C8—S1—C7	0.3 (2)
C25—C26—C27—C22	1.5 (4)	C14—C7—S1—C8	0.1 (2)
C25—C26—C27—C28	−175.3 (3)	C6—C7—S1—C8	178.8 (3)
C23—C22—C27—C26	−1.1 (4)	C23—C22—S2—C21	−177.0 (3)
S2—C22—C27—C26	−179.8 (2)	C27—C22—S2—C21	1.7 (2)
C23—C22—C27—C28	176.3 (3)	C28—C21—S2—C22	−0.5 (2)
S2—C22—C27—C28	−2.5 (3)	C20—C21—S2—C22	177.5 (3)

### Hydrogen-bond geometry (Å, º)

Cg4 and Cg14 are the centroids of the C8–C13 and C22–C27 rings, respectively.

**Table:**

*D*—H···*A*	*D*—H	H···*A*	*D*···*A*	*D*—H···*A*
C20—H20*B*···O2^i^	0.97	2.48	3.307 (4)	144
C3—H3*B*···*Cg*14	0.97	2.59	3.502 (3)	157
C17—H17*A*···*Cg*4	0.97	2.92	3.800 (4)	151
C29—H29*B*···*Cg*4^ii^	0.97	2.90	3.706 (3)	142

Symmetry codes: (i) −*x*+1, *y*+1/2, −*z*+2; (ii) *x*−1, *y*, *z*.
